# FRIENDSHIP QUALITY IN COLLEGE PREDICTS WELL-BEING IN QUALITATIVE LIFE REFLECTIONS 50 YEARS LATER

**DOI:** 10.1093/geroni/igae098.2223

**Published:** 2024-12-31

**Authors:** William Chopik

**Affiliations:** Michigan State University East Lansing, East Lansing, Michigan, United States

## Abstract

Theories of successful aging and psychosocial development posit that well-being later life indicates successful adaptation and portends a longer life. In identifying antecedents to late life well-being, close relationships have emerged as a prominent antecedent for health and well-being across life. However, prospective studies examining the impact of social relationships on health and well-being are relatively rare, particularly mixed methods studies. I paired the Harvard Student Study Class of 1964 (N = 252) with qualitative reflection data in their alumni class reports every five years after graduation for over fifty years. Machine learning techniques will be featured depicting how psychological traits can be inferred through autobiographical reflections. Having more friends and closer friends during their freshman year of college (at age 22) was associated with more positive life reflections (r =.22), more positive relationships (r =.23), and less mental illness (r = -.25) fifty years later (at age 72). I will then leverage these archival sources to examine the impact of social relationships on health and well-being across nearly 100 years to test secular changes in a newly constructed cohort sequential design. Emerging methods for linking family reports across generations and cohorts will be discussed and preliminary results presented. The discussion of the talk will focus on the antecedents of successful aging and how to leverage existing data—in its many forms—to study psychosocial development within a person’s life and across historical time.

